# Appealing to Tacit Knowledge and Axiology to Enhance Medical Practice in the COVID-19 Pandemic: A Systematic Review and Hermeneutic Bioethical Analysis

**DOI:** 10.3389/fpubh.2021.686773

**Published:** 2021-12-08

**Authors:** Ana-Beatriz Serrano-Zamago, Myriam M. Altamirano-Bustamante

**Affiliations:** ^1^Grupo Transfuncional en Bioética, Centro Médico Nacional Siglo XXI, Instituto Mexicano del Seguro Social, Ciudad de México, México; ^2^Master and Doctorate Program in Medical and Health Sciences, Universidad Nacional Autónoma de México, Mexico City, Mexico; ^3^Escuela Superior de Medicina, Instituto Politécnico Nacional, Ciudad de México, México; ^4^Unidad de Investigación en Enfermedades Metabólicas, Instituto Mexicano del Seguro Social, Ciudad de México, México

**Keywords:** medical ethics, value, philosophy of medicine, practical reasoning, COVID-19 pandemic, tacit knowledge

## Abstract

**Background:** The pressure of coronavirus disease 2019 (COVID-19) pandemic, epidemiological and demographic changes, personnel-patient relationship in healthcare, and the development of biotechnologies do not go unnoticed by the healthcare professional. Changes are so wide and at a high rate that guidelines and mere scientific knowledge, which are represented by evidence-based medicine, are not sufficient to lead actions, thus the experiential aspects in the configuration of an ethos present as a fundamental part of the resources to deal with critical scenarios, such as a pandemic. In this regard, the recognition of tacit knowledge as a way of teaching and learning skills related to ethical aspects such as principles, virtues, and values, revealed as a fundamental part of the clinical field. The challenge is to strengthen binomial evidence-based medicine and values-based in order to achieve excellence in the health care of the patients and the well-being of the clinical personnel.

**Method:** A 2-fold analysis was conducted taking pediatric endocrinology as an example. First, a systematic review was carried out in electronic databases BIREME, PubMed, and PhilPapers following PEO and PRISMA approaches. A total of 132 articles were garnered. After reading their title and abstract, 30 articles were obtained. Quantitative information was arranged in an Excel database according to three themes: ethics, axiology, and tacit knowledge. A quality criterion that meets our research question was assigned to each article and those which had a quality criterion of 3 (9) were taken to carry out the hermeneutic bioethical analysis, which consisted of three stages, namely naïve reading, codification, and interpretation. The results were analyzed in Atlas.ti. to elucidate the relations between the three main themes in accordance with the objective.

**Results:** Although there was no difference in the frequency of tacit knowledge skills, including cognitive, social, and technical, for medical practice, there is an intrinsic relationship between epistemic and ethical values with cognitive skills, this means that professionals who practice honesty, authenticity and self-control are capable of seeing patients as persons and thus respect their dignity. This suggested that there is a strong partnership between evidence-based medicine and value-based medicine, which reinforced this binomial as the two feet on which medical practice decisions rested. With regard to tacit knowledge in terms of the context of the COVID-19 pandemic, the challenges refer to (1) adapting and learning a new way of establishing trust with the patient and (2) how to capitalize on the new knowledge that new experiences have posed.

**Discussion:** The analysis of ethical-tacit knowledge in medicine is a recent phenomenon and is in full development. Although no references were found that dealt with any of the main topics in pediatric endocrinology, there is an interest in pediatrics to explore and discuss educational strategies in ethics related to its tacit dimension as a vector of enhancement in the clinical practice. Educational strategies ought to take into consideration the development of skills that promote reflection and discussion of experiences, even more vigorously in the context of the COVID-19 pandemic.

## Introduction

The flow of clinical practice amid the coronavirus disease 2019 (COVID-19) pandemic, like all human activities, finds itself constantly updating due to the influence of intrinsic factors coming from personal and professional challenges and extrinsic factors stemming from the history of life and the social, cultural, environmental, and pandemic contexts (1, 2) the political and economic climate also influence (3). Historically, health care systems, which require the collaboration of cross-functional groups, have been structured in diverse ways in an attempt to solve problems, challenges, and needs that arise from the health/disease process (4).

The pandemic brought by COVID-19 has called healthcare practice for a reevaluation of the configuration of knowledge upon which it builds itself. In this day and age, the pressure of global and local changes going on in epidemiology, demographics, and healthcare personnel-patients relationship is triggered by the accelerated biomedical and biotechnological advances (5–10).

The kind of knowledge needed to face these issues has to consider the indissolubility of the relationship between scientific aspects and ethical ones that play a meaningful role in the configuration of the patient-physician relationship—the two feet in decision-making (11), that is, the training of experts in biotechnologies as well as agents who, coming from an education stemmed in values, are capable of evaluating the contexts in which they perform (practical knowledge) and can develop skills to interact with others (patients and healthcare teamwork) (11–19). In this sense, the learning process presents itself as a fundamental resource, raising the need for a reflection about the type of knowledge that (1) spreads in the clinical practice environment and (2) comes into play in decision-making. However, not much attention has been paid to the development of teaching-learning strategies.

In an extensive sense, two ways of knowledge acquisition and performance are recognized: a formal one that we learn and express explicitly through guidelines and that works in a focalized mean, while the other alludes to a tacit dimension (20), which remains in the edge of attention and incorporates aspects that are taught and learned mostly through practice and in a comprehensive manner (it is context-specific) (16). Both routes of knowledge come into play when performing an action, the first allows us to pay attention to the main object of our action while the second is full of details that we give for granted and nevertheless makes the difference between a novice and a skillful performance.

Carrying out a diagnostic is for clinical practice the main example of the development of skills, which mostly consist of forming abilities or “specialized knowledge,” which may cross disciplines very well and which can also be acquired through observing explicit rules and norms as well as from personal training or rapport with someone of greater skill (20). Personal experience adds as a means of knowledge acquisition, not only going through it but also its observation and sharing, where a system of values and rules that are not made explicit arise.

The relevance of tacit knowledge in the general construction of knowledge has come a long way since Polanyi (20) first noted it, extending to different fields in the search for an improvement of their practical skills.

When the physician conducts auscultation the focus is in the search of signs that may integrate together with the patient's symptoms and lead to several pathological syndromes and does not pay focused attention to other details that nevertheless allow to carry out such activity. Depending on the degree of expertise will be the elements the doctor will pay attention to. For example, unlike a novice, an expert will select what to ask according to the patient's answers and will not travel the complete series of questions that he probably learned in classes that should be asked in a diagnostic interview. In addition, he will pay less attention to the way in which he manipulates the diagnostic instruments, for instance, in the case of an ophthalmologist, the ophthalmoscope, and will be perceived by the patient with dexterity or skill in his handling. He will even know how to ask for a posture and ideal movements to make a better examination (21).

Another example of the path that explicit and tacit knowledge follows to come into action in medicine is patho-clinical sessions, where cases are reviewed and discussed by comparing them with nosological diagnoses. This exercise forces the physician to look back into the processes that lead to diagnosis and state those hidden details for others (22, 23).

As a result of seeing patient after patient, the physician acquires a type of knowledge that is tacit and personal, which allows the development of prototype cases to use in the future and also feedback on those which are learned explicitly (21). This type of knowledge is a tool to organize information and act in specific situations, even to interact with others—patients and work team- in an ethical and humanistic manner. In this work, ethics will be considered as the reflection on one's own actions and behaviors directed toward the best way to live life.

In this sense values are a fundamental part of ethical knowledge, which are at the core of the action in clinical practice and are tacitly transmitted. These will be understood as resources used as guides that allow us to consider actions, objects, or situations as good, desirable, pleasing, convenient, or useful toward certain ends (3). These ends and the values which guide them allow the development of a sensibility toward our own lives and professional practices (3, 4), which are handed down generation to generation, specifically down to doctors-to-be.

The attention paid to this kind of knowledge will help to generate awareness about the ethical-tacit contents that underlie decision-making and clinical encounter that is furthermore shared in the teaching-learning process. This can trigger the proposition of more suitable content for nowadays clinical practice and teaching-learning strategies that will surely result in the reduction of complaints and burn-out syndrome. In the context of a pandemic, scientific knowledge, usually represented by evidence-based medicine, is not sufficient to face complicated decision-making. The development of a values-based medicine is needed to consider comprehensively the patient.

Nevertheless, its relevance, the appreciation of ethical-tacit knowledge (ETK) in clinical knowledge (24–26) and practice (27–29) is a recent phenomenon, currently developing. In this paper our main objective was to explore the literature that touches on ethical tacit knowledge in the medical sphere to make a sound description and analysis of its elements to understand the impact it has on the flourishing of doctors-to-be in the context of the COVID-19 pandemic, taking pediatric endocrinology as an example given the relevance of the care provided by this specialty throughout the life of individuals. To achieve this objective the specific goals were the following: to find out the ways in which articles have reached the identification of ethical tacit knowledge, how is perceived its convenience in the specific domain of medicine, and how they describe it. Derived from the aforementioned objectives, the following questions were raised:

1. How is identified, perceived, and described ethical-tacit knowledge in the medical sphere, for example, in the field of pediatric endocrinology in the context of the COVID-19 pandemic?
2. What is the impact ethical-tacit knowledge having on the flourishing of doctors-to-be?

To achieve the objectives and answer the questions, a two-fold analysis was performed: first a systematic review following participants, exposition, and outcomes (PEO) and preferred reporting items for systematic reviews and meta-analyses (PRISMA) approaches and secondly a hermeneutic bioethical analysis. The amalgam of both analyses promoted the deep understanding of the pathways by which knowledge (as the disposition to act), specifically ethical tacit one, is built-in clinical practice. Informing the medical community about these results can help to promote their everyday labors and decision making which in consequence might encourage the academic development of a new generation of health care professionals.

## Methods

A systematic review of scientific literature was carried out to obtain original articles, particularly in fold one, and then perform a hermeneutic-bioethical analysis (30–32). This study was carried out according to the PRISMA declaration (32) and a modified PICO approach, namely PEO Strategy. There was no need for previous ethical approval or informed consent.

### Fold One: Systematic Review

#### Study Characteristics. PEO Strategy

Participants, exposition, and outcomes (PEO) Strategy (modified from PICO Strategy) addresses the research questions. PEO represents an acronym for Participants, Exposition, and Outcome. These three components make reference to the essential elements that helped to construct the MeSH (Medical Subject Headings) terms used in this work and guided the study research. The description of the PEO Strategy is as follows:

P (Participants): medicine, clinical practice, physician, medical doctor, pediatrician, endocrinologist,
E (Exposition): application of instruments (surveys and interviews) and participant observation,
O (Outcomes): development of an axiological profile (characterization of the value system) or a model about the characteristics of ethical tacit knowledge.

#### Database Search

The systematic search was carried out according to the PRISMA declaration (32) in electronic databases BIREME (Centro Latinoamericano y del Caribe de Información en Ciencias de la Salud de la Organización Panamericana de la Salud), PubMed (National Library of Medicine), and PhilPapers (Philosopher's Information Center). All references were organized and saved in Mendeley to eliminate duplicates. Articles in English and Spanish were selected with no year limit. The sources were searched and recollected in august 2020.

#### Eligibility Criteria

The search was made following de decision tree designed with de medical subject headings (MeSH) terms obtained from the PEO strategy. The intention was to find articles that helped to achieve the objective by making a relationship between the selected participants, research strategies, and expected outcomes. Duplicates were removed and a total of 132 articles were garnered.

Both authors independently screened each record to make sure they met the criteria of the review and then compared their results.

Thereafter, we conducted a screening and categorization in our Mendeley database (Free reference manager software) following our objective and according to three major themes. We specifically searched for: ethics, axiology (values system), and tacit knowledge. The articles were divided into three themes to differentiate their focus or accents. Since for “tacit knowledge” no results were obtained, a manual search was carried out using more general MeSH terms: medicine, tacit knowledge, and ethics, in different combinations. The results obtained were 14 relevant articles for ethics, 5 for axiology (values), and 11 for tacit knowledge, after reading the title and abstract.

#### Quantitative Information

Subsequently, the entire text was read (30 articles) to gather the following quantitative information: bibliographic information (1st author and year), setting, main school (s) of thought or concepts, study design/intervention, target population, among other aspects that were specific to the three themes: ethics, axiology (values) and tacit knowledge. All the obtained information was arranged in an Excel database.

A quality criterion was assigned to each article to assure the soundness of the methodology (32). Three aspects were taken into consideration: a clear objective, a clear methodology, and clear and relevant discussions and conclusions; each aspect added a point, considering three as maximum.

Fold one (systematic review) helped to draw a panorama on how tacit knowledge is considered, described, and identified in the medical spheres and which is its ethical substance.

A complementary search was carried out to incorporate articles that relate tacit knowledge and the COVID-19 pandemic. The purpose was to show the relevance of the implementation of the three main topics of this article to the emergency situation posed by the de COVID-19 pandemic.

### Fold Two: Hermeneutic-Bioethical Analysis

The hermeneutic study is used as a means to interrogate the text, knowledge contents and thus achieve an interpretation of it based on profound comprehension, in this case of ethical and bioethical issues (axiological content in relation to the comprehensive development of the being) related to the clinical practice and tacit knowledge within. We followed the hermeneutical analysis as proposed by Paul Ricoeur (31). Said analysis is a dialectic process between the whole and its parts, ranging from comprehension to explanation; it allows for a better and deeper understanding. Besides, as Geanellos (30) mentions, hermeneutics is an advantage in research to achieve congruence between philosophy, methodology, and method.

The analysis consisted of three stages: first, a naïve reading that allows for a holistic and general comprehension; second, a coding and division of the elements to identify relevant content; and the third is an interpretation which is achieved by incorporating all of the elements described previously: the naïve reading, the structural analysis, the research questions, and the presented grounds.

The articles that resulted from the systematic review with a quality criterion of 3 (nine articles) were taken to carry out the hermeneutic bioethical analysis. The first stage of naïve reading refers to giving an open-minded look at the articles to have a general understanding and starting to think about classifications.

Afterwards, to develop the second stage of hermeneutic analysis (structural analysis), the content of the articles was codified and divided into families, which were then separated into topics and subtopics with the help of the Atlas.ti. program (Qualitative data analysis and research software). The families were defined by the three main themes, namely ethics, axiology, and tacit knowledge. Ethics was organized into one topic and four subtopics which make reference to ethical schools that are usually cited in the general bibliography. In regard to axiology, this family was subdivided into two topics and eight subtopics. The topics and subtopics were chosen according to an observed tendency in the axiological horizon identified in the systematic review and analyzed taking into account the perspective developed by Echeverría (33). This author starts from the idea that the paradigm of technoscience, where there is no scientific progress without a technological advance-,floods all human activities, including clinical practice, and that the complex system of values that integrate its axiology can be analyzed through a series of subsystems that interact whit each other. The third family, namely tacit knowledge, was divided into three topics and 16 subtopics using the work of Gary Insch and collaborators as reference (34). They developed an instrument to measure tacit knowledge in college students from three dimensions of skills, specifically cognitive, technical, and social. The complete families, their topics, and subtopics can be consulted in Table 1.

**Table 1:** Ethics, Axiology, and Tacit knowledge codes to perform the qualitative analysis in Atlas.ti.

**Family**	**Topic**	**Subtopic**
1. Ethics	1.1 Schools of thought	1.1.1 Utilitarianism 1.1.2 Deontology 1.1.3 Aretology 1.1.4 Principlism
2. Axiology	2.1 Epistemic Values	2.1.1 Authenticity 2.1.2 Accuracy 2.1.3 Coherence
	2.2 Ethical/ Bioethical Values	2.2.1. Life care 2.2.2 Respect 2.2.3 Liberty 2.2.4 Honesty 2.2.5 Dignity of the human person
3. Tacit knowledge	3.1 Cognitive Skills	3.1.1 Self-organization 3.1.2 Willingness to learn 3.1.3 Heuristic ability 3.1.4 Introspection 3.1.5 Self-control 3.1.6 Complete activities on time
	3.2 Technical Skills	3.2.1 Master the use of instruments 3.2.2 Find the most appropriate method 3.2.3 Combines own time with the institutional 3.2.4 Joint discussion of results 3.2.5 Arranges meetings with superiors/area managers
	3.3 Social Skills	3.3.1 Ability to listen to peers (health staff) 3.3.2 Ability to listen to non-peers (support net, family, patient) 3.3.3 Joint and transdisciplinary work 3.3.4 Networking 3.3.5 Share experiences
4. Emerging codes	4.1 Dilemmas 4.2 Confidence	

It is worth mentioning that two other codes were added, namely, dilemmas and confidence, to the ones previously described in Table 1, which emerged as relevant at the time of coding.

## Results

Articles were thoroughly revised by two of the authors of this present article to ensure that the selection of codes coincided with the meaning of the topics and subtopics.

### Fold One: Systematic Review-The State of the Art

**Figure 1:**
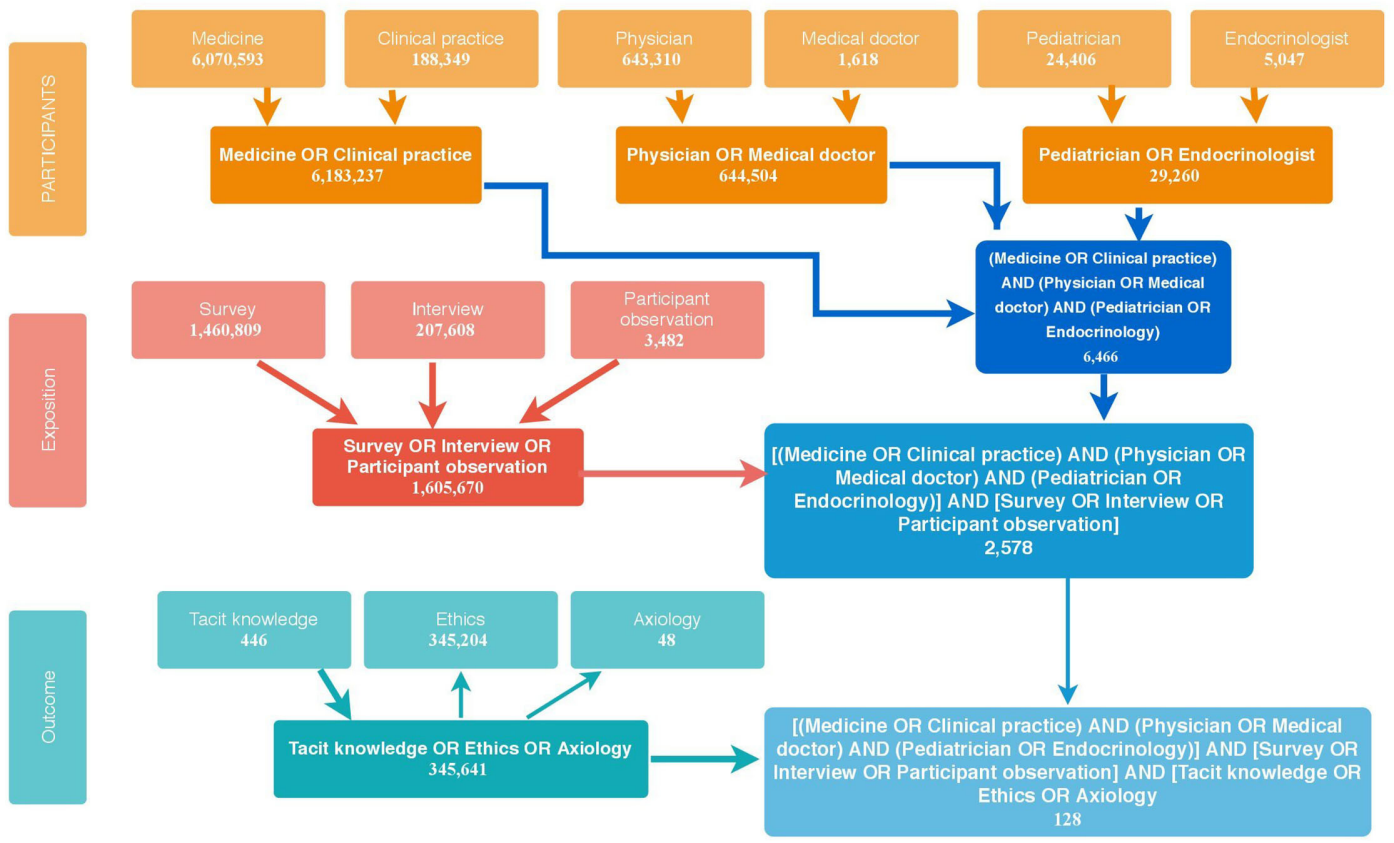
PubMed decision tree.

The search strategy yielded a total of 132 articles. The MeSH terms used according to the PEO strategy and the decision tree for the search can be found in Figure 1, where the search details used in PubMed are represented.

On the vertical axis, it is possible to observe the three planes of the PEO strategy, namely, participants, exposition, and outcome, and on the horizontal one, the Boolean operators used to relate each of the planes within and between them. This entire process was carried out in order to obtain the most suitable MeSH term to search for articles in the rest of the databases. The precise description of the search results of the MeSH terms can be consulted in Supplementary Material 1.

**Figure 2:**
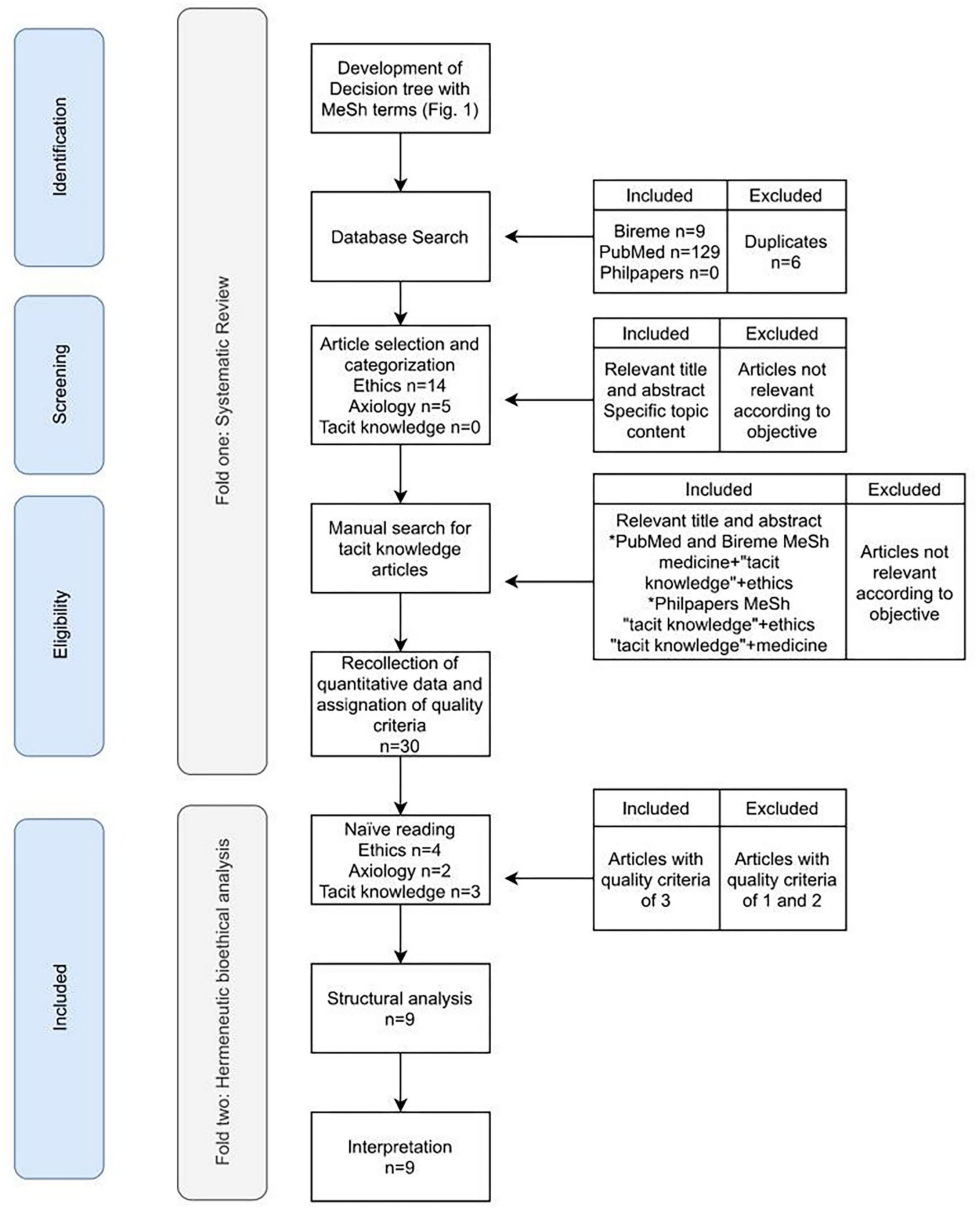
Fold One and Fold Two Flowchart according to PRISMA.

To carry out Fold One, which includes the search in both PubMed and the other databases mentioned in the Methodology, the steps described in the PRISMA statement were followed -identification, screening, and eligibility. These are indicated in the text boxes at the extreme left of Figure 2, while the inclusion and exclusion criteria used in each step are described in the boxes on the extreme right and the results of each one are described in the central boxes of the figure. The identification stage refers to the search for articles in all databases using the MeSH term that was obtained through the PubMed decision tree, then a screening was performed to make a selection of articles and divide them according to the three topics of interest of this work -ethics, axiology, and tacit knowledge. Finally, the eligibility step was performed to collect quantitative data from the selected articles and assign a quality criterion to continue the analysis.

The complete quantitative information by topic can be consulted in Supplementary Materials 2–4. In general, the name of the author and the year of publication, the study location, the study design, and the target population were obtained, and the quality index was assigned. In addition, specifically for the topic of ethics, the main school (s) of thought or concept (s) and a description of the epistemic subject were collected; for axiology, the theoretical axis of each article was recovered; and for tacit knowledge the theoretical axis, tacit knowledge conception, tacit knowledge identified relevance and the medical fields related were captured.

We observed a differentiated pattern according to the setting of research for the three main research topics of this article: ethics, axiology, and tacit knowledge. To describe it, geographical categories were arranged taking into consideration the historical and philosophical traditions of the five Continents: two for the Americas (AA-Anglo-Saxon America and LA-Latin America), six for Europe (I-Iberia, WE-Western Europe, EE-Eastern Europe, NC-Nordic Countries, SE-South Europe, and the UI-United Kingdom and Ireland), three for Asia (EA-East Asia, WA-West Asia, SA-South Asia), one for Oceania (O) and one for Africa (Af). Complete information can be found in Table 2.

**Table 2:** Regions of articles publication according to the three main themes.

**Region**	**Initials**	**Ethics**	**Axiology**	**Tacit knowledge**	**Total**
Anglo-Saxon America	AA	6	2	11	19
Latin America	LA	1	7	1	9
Iberia	I	0	0	0	0
Western Europe	WE	1	0	1	2
Eastern Europe	EE	0	0	0	0
Nordic Countries	NC	2	1	2	5
South Europe	SE	0	0	1	1
United Kingdom and Ireland	UI	0	1	2	3
Eastern Asia	EA	0	0	1	1
West Asia	WA	0	0	0	0
South Asia	SA	0	0	1	1
Oceania	O	0	0	0	0
Africa	Af	0	0	0	0
**Total**	—	10	11	20	**41**

#### Ethical Decisions as a Matter of Education and Experience

**Figure 3:**
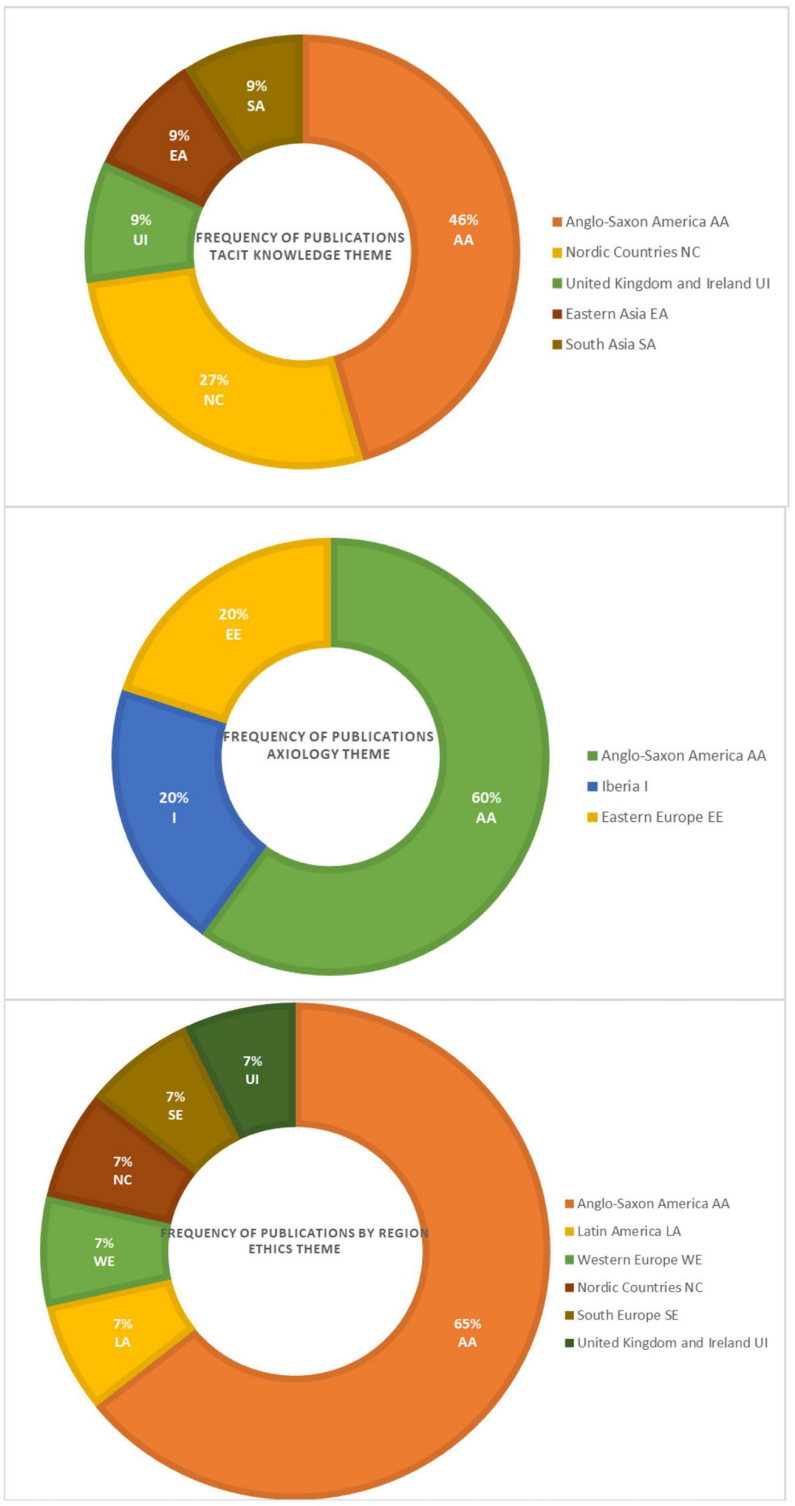
Article's production by region and the main theme.

Regarding the ethics theme, the production of articles was dominant in more than 50% of the AA region (Figure 3). Then we found in equal percentages publications in LA, WE, NC, SE, UI. In AA the discussions touched on matters related mainly to decision making regarding end-of-life (35), the development of communication skills with patient-parents to take shared decisions (36), and the search for ethical assistance by the medical team (37). Reflection and discussion as a means of confronting ethical challenges (38, 39) are also mentioned, as well as aspects related to ethics education (40), professional autonomy (41), and conflict of interest when writing clinical practice guidelines (42).

Meanwhile, in LA, a relationship is made between ethics education and the promotion of reflection on professional attitudes (43). In WE, the accent is given to the relevance of taking shared decisions in Pediatrics and acknowledging differences in ethics education, patient care focus, and exposure to the patients to avoid conflicts when making decisions.

In NC the ethical reasons related to the background of physicians that lead the relationship with patients and which results in weighing the autonomy of the patient or a paternalistic attitude are questioned (44). In SE the experience of practice and education of physicians is also described to be related to the balance that is made between taking a paternalistic attitude and respecting the patient's autonomy (45).

In UI, the complexity of social processes is rather analyzed from the perspective of the usefulness and reception of a technological device to make daily tasks easier in a hospital environment (46).

#### What Is Valued: The Axiological Cornerstone

In relation to axiology, production was concentrated in the AA area by 60%, followed by regions EE and I with 20% (Figure 3). In AA, the discussion about values revolved around honesty when disclosing errors (47), peer discussion in decision-making (48), and satisfaction with the profession (49). It can be seen that each of the articles covered a different level in the depth of reflection of the health care personnel: toward others (the patient-tutor dyad), toward colleagues, and toward themselves. In EE the paternalistic role of pediatricians is reviewed in the light of the value of life, as a cultural value (50) while in Iberia the value of pediatrics is highlighted from primary care, which should be practiced jointly and intersectorally as part of supporting activities not only for health promotion but also for education and prevention (51).

It is worth mentioning that despite the fact that what was sought in the revised articles was the description of the value system of a medical specialty such as pediatrics, apparently there is no such development. Rather, what was obtained was a reflection on what is valued in the field of this specialty, such as joint work in decision-making and the recognition of experience as a key factor in it, as above mentioned.

However, it is worth mentioning the identification of the value of honesty as a core value when pediatricians confront the decision to disclose an error either to parents or patients (47). Such a decision becomes complicated for physicians as the repercussions of such an act can cost them both a legal claim and the reputation among their colleagues and work team. Therefore, they are most inclined to disclose an error only when it is most apparent to the parents. Although Loren et al. (47) mention ideal actions, specifically disclose the error, provide all the details, offer an explicit apology, and discuss detailed plans to prevent future recurrences, they also account for the lack of information, training, and support that exists among physicians to discern which means are best suited to the good they seek in certain circumstances, namely, *phronesis*.

#### An Individual Knowledge That Can Be Collectivized

On the topic of tacit knowledge, the production of the articles was concentrated in the AA region in a little <50%, followed by NC in 27%, and finally in equal percentages in UI, EA, SA (Figure 3). In AA, tacit knowledge is considered relevant both in the public sphere for planning programs based on shared experiences and interactive conversation (26), and in the private sphere as an element of professional identity, and therefore relevant for the construction of the being (52). Discussions on this topic are perceived as relevant for addressing intuitive aspects of clinical reasoning (25, 53, 54). In the second region with the highest percentage, NC, including the discussion on tacit knowledge to reflect on medical practice allows identifying what is necessary to understand and act in specific situations (55) according to the context of the patients and the specialty, as well as to develop (through a process of explicitation) communication skills (56, 57). In UI, its usefulness in the construction of good judgment is theoretically discussed (28) while EA and SA propose and test educational strategies to share a type of knowledge that is practical, either through videos (58) or in the planning of online strategies to enrich problem-based learning (59).

#### Methodological Approaches to Ethical-Tacit Knowledge in Medicine

**Figure 4:**
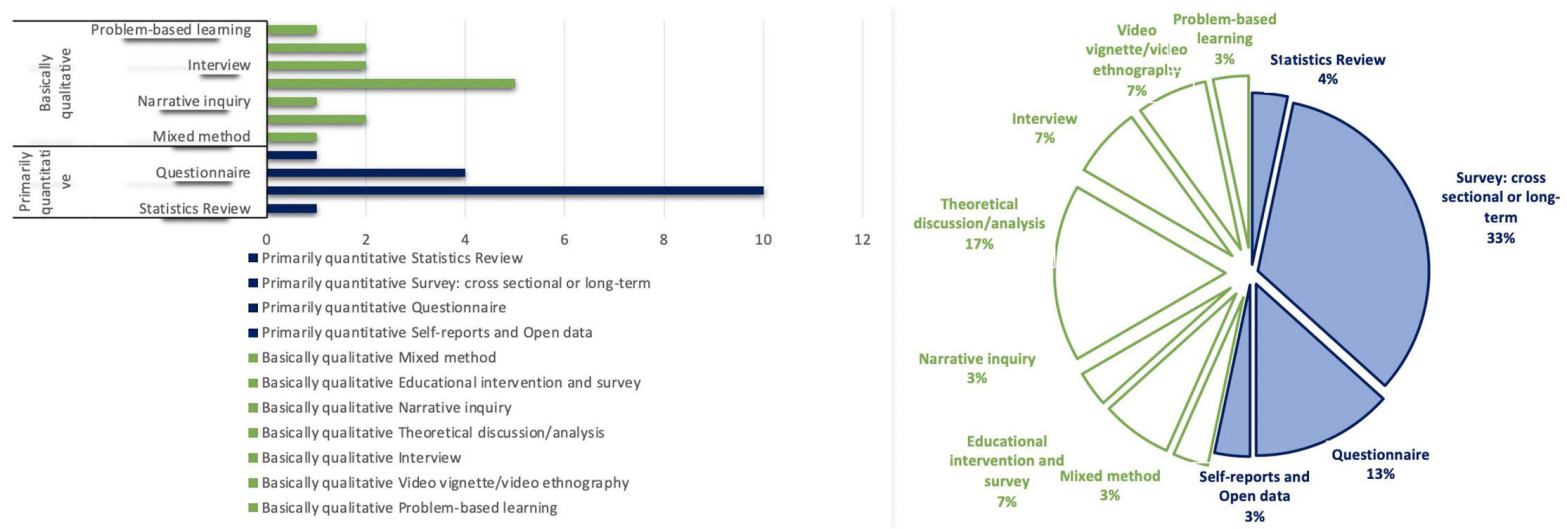
Methodological approaches to tacit knowledge.

In this paper, there was an interest in showing up the variety of methodologies used to elucidate the nature and scopes of ETK in medicine. The different methodologies were organized into two sorts. On the one hand, there are those characterized by a quantitative approach, such as questionnaires, surveys, or the review of data and statistics. On the other hand, there are those that have a qualitative perspective such as interviews, educational interventions, and those that make an analysis of narratives or an in-depth theoretical discussion. Figure 4 shows that the most widely used resources to explore ethical tacit knowledge are surveys. Theoretical discussion or analysis comes in second place, and in third place are questionnaires. Meanwhile, educational interventions, interviews, and video ethnography come in fourth place equally, followed by statistics review, self-reports, mixed methods, narrative inquiry, and problem-based learning.

Although quantitative methodologies were used more frequently (52%), the difference between these and qualitative ones (47%) was not that great. Questionnaires and surveys, which belong to the quantitative approach, were the methodologies most chosen to work on the subject of ethics and axiology, while the preferred approach to address the subject of tacit knowledge was the qualitative one: almost 50% theoretical discussions/analysis and the remaining percentage varied among the other qualitative methodologies. This is in line with one of our expectations since we come from the notion that qualitative methodologies (or mixed) have better tools to unravel the content and the meaning of tacit knowledge that is put into practice in everyday activities.

**Figure 5:**
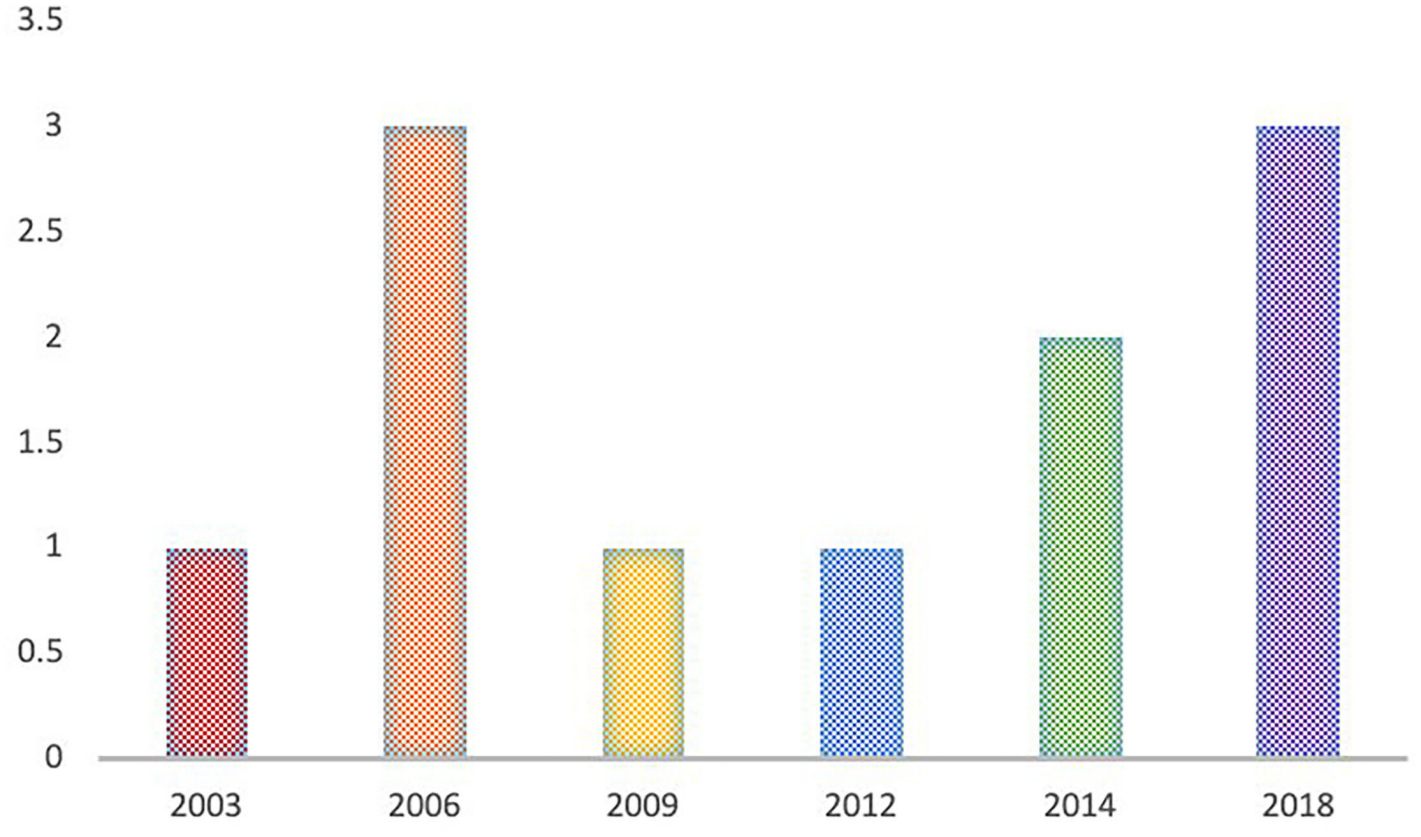
Annual production of articles for tacit knowledge theme.

Regarding the topic of tacit knowledge, another aspect that was reviewed was the annual production of articles to find out if any pattern could be recognized. However, this was not the case, the highest production (28%) was found in 2006, from there it jumps to 2018 (27%), followed by 2014 (18%). Although over time there is an increase in production regarding the subject of tacit knowledge, it is not constant (Figure 5).

#### Shaping the Gears of the Machinery: Conceiving Tacit Knowledge

From the definitions in Supplementary Material 4, it is possible to take concepts that appear in such a constant way outlining the characteristics that, according to the reviewed articles, describe tacit knowledge.

**Figure 6:**
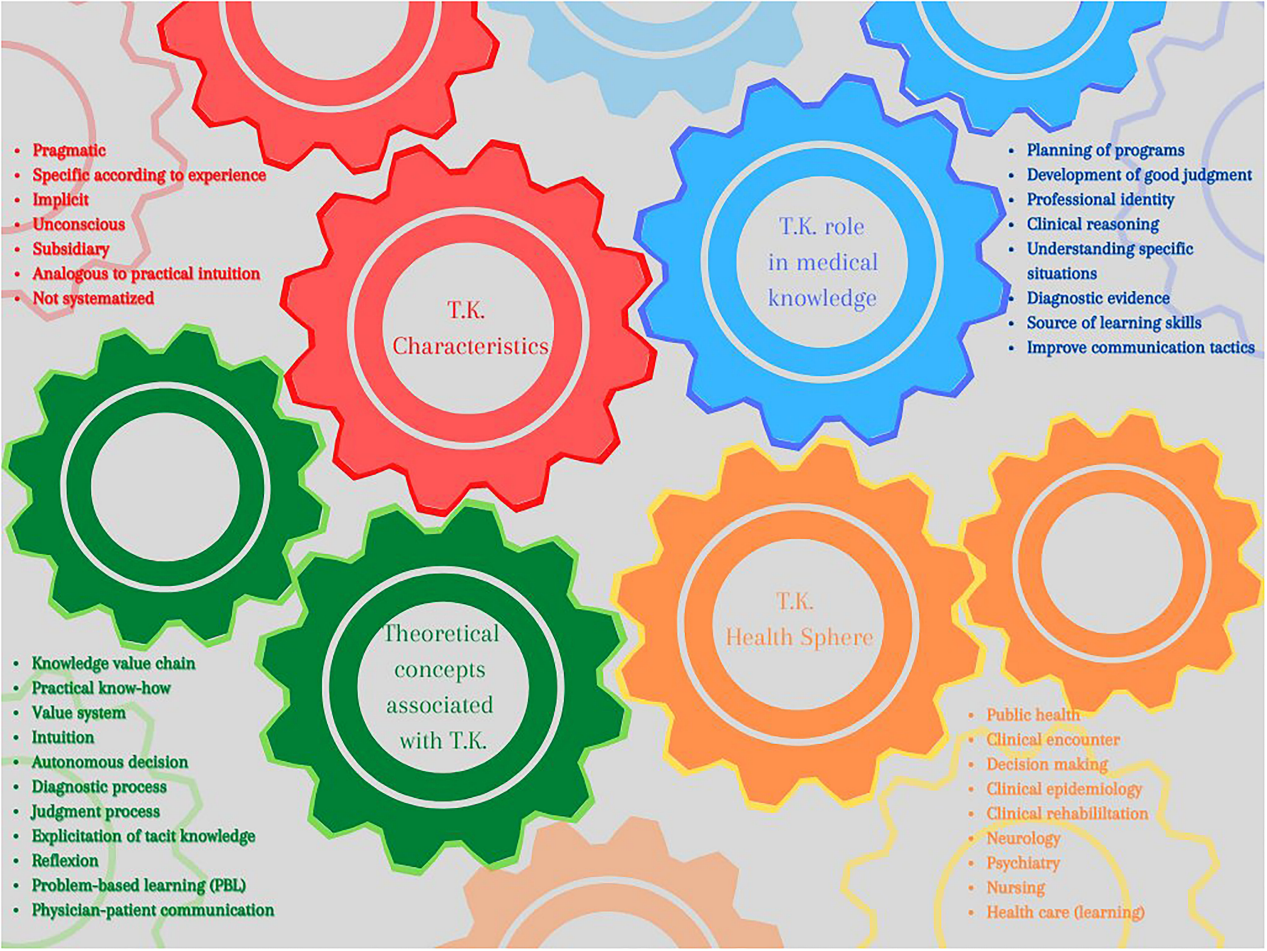
The gears of T.K. machinery.

Seven main characteristics were found (Figure 6):

(a) Unconscious: tacit knowledge is referred to as a kind of knowledge that acts in such a way that we are not aware of it, thus it is hard to account for (52, 54).
(b) Implicit and (c) Not systematized: it comes into play when making a judgment but cannot be stated; it goes beyond explicit guidelines and codifications outlining a good practice (28, 57).
(d) Subsidiary: speaks of the relationship that tacit knowledge and explicit knowledge hold when actions are to be taken, considering the first as peripheral or subsidiary and the second as main or principal (25).
(e) Specific according to experience and (f) Pragmatic: it is the knowledge that is developed from particular experiences and repeated actions (26, 56, 58).
(f) Intuitive: given its unconscious and unspecifiable character, tacit knowledge has been described as a mechanism that acts through intuition (25, 59).

Now, due to the characteristics described above, there are several theoretical concepts that can be linked with tacit knowledge to deepen and guide its study. Some of these are associated with evaluative issues (52, 60) (value chain and value system), others with processes (54–56) (in decision-making, to carry out diagnoses and judgments, and make tacit knowledge explicit) and others with skills learning and development (57–59) (reflection, intuition, physician-patient communication, and problem-based learning) (Figure 6).

All these characteristics help to make a description of tacit knowledge land even more specifically in the field of medicine. Among the articles reviewed on the topic of tacit knowledge, those that made a theoretical analysis focused on general areas such as decision-making, clinical encounter, and learning about health care. The rest of the articles addressed spheres such as public health, epidemiology, rehabilitation, neurology, psychiatry, and nursing (Figure 6).

For the fields previously mentioned, tacit knowledge is seen as an aid to reach expertise in practical aspects, as well as exploring subjective content in communication. This opens the possibility of developing good judgment and, therefore reduces prejudice through the exploration of own behaviors in practice (Figure 6).

#### Highlighting Pediatrics

Among the quantitative information, the description of the target population was obtained in order to know how often pediatrics and its subspecialties, such as pediatric endocrinology, are clearly mentioned.

It is noteworthy that the specialty of pediatric pediatrics is mentioned for the ethics and axiology themes of interest, but nothing regarding tacit knowledge (SP 4). Among the pediatric subspecialties that are most reviewed are neonatologists and intensivists since both are clearly related to complex decision-making at the beginning of life and because of the danger of death. However, pediatric pharmacists, primary care in pediatrics and the formative stages of the specialty in residency are also integrated into the discussions.

### Fold Two: Hermeneutic-Bioethical Analysis

In this second stage of analysis, the purpose was to deeply understand the relations between tacit knowledge, axiology, and ethical perspectives.

#### Relationships Between Schools of Ethical Thought, Tacit Knowledge, and Axiology

**Figure 7:**
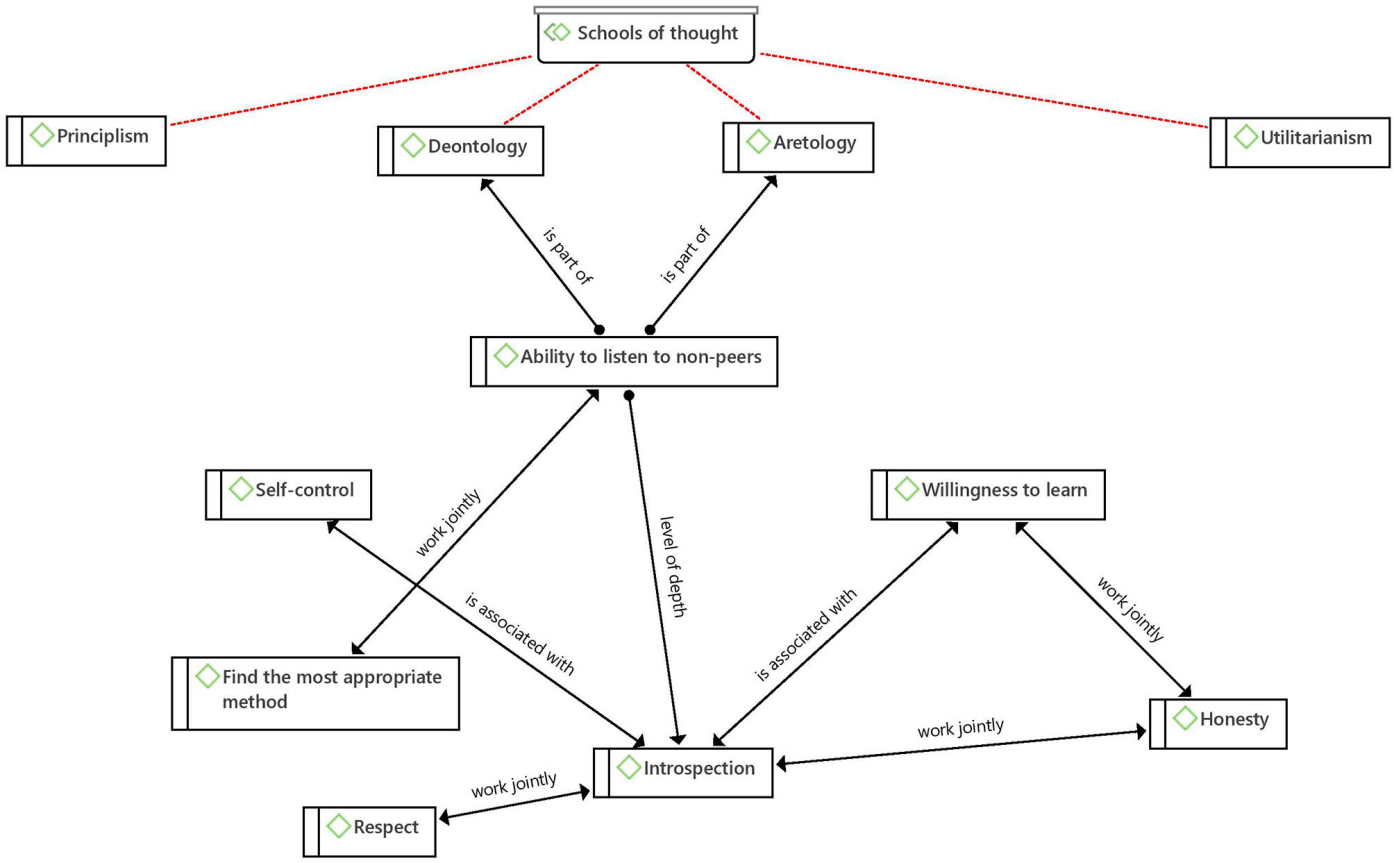
Net between ethical schools of thought.

Among the four schools of ethical thought that were sought, the one more strongly identified was the deontological, followed by the aretological, which have as union point the Ability to listen to non-peers -as shown on Figure 7's net- a social skill that reveals as part of the duty of the medical profession to achieve joint decision-making and as a means to cultivate humility and virtuous life by taking the others into consideration -their beliefs, concerns, and interests:

“The assessment was a really complex procedure. The findings show that these physicians used both a biomedical approach through medical procedures and a holistic approach where they used a broad palette of information about the person, the environment (including both the work and home environment) and tasks/occupations at work” (56).

This is the path to reach another level of reflection and awareness of one's own actions, namely introspection, which is a cognitive ability supported by other skills of the same type and associated with bioethical values such as honesty and respect.

Introspection allows an analysis of one's own good behavior, which includes making an honest evaluation of oneself and from there acting with respect for opinions and comments different from one's own -from colleagues, patients, and in the case of pediatrics, parents of patients—to know how to incorporate them into the clinical event:

“…it was emphasized that initially the patient should present her goals and tell about how far the goals from the former meeting had been achieved. Then the different practitioners commented on the situation and on the realism in the patient's goals. It was seen as important that the patient's true hopes and wishes were expressed and respected” (55).

#### Axiological Associations

**Figure 8:**
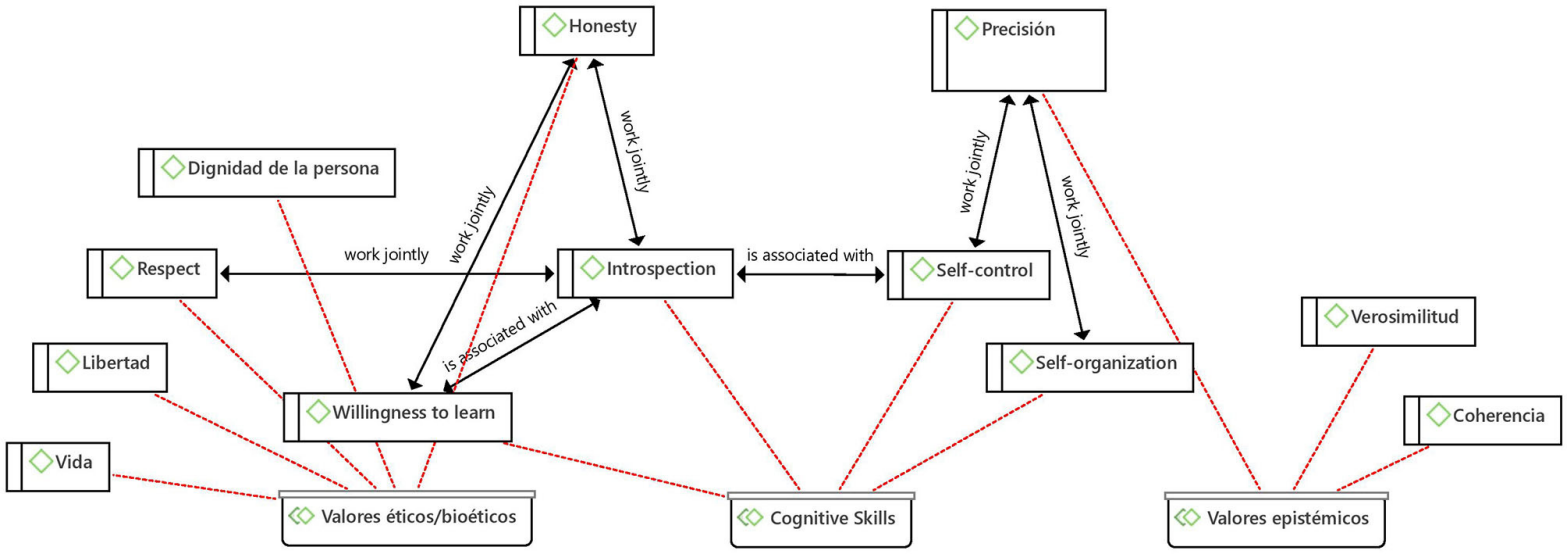
Net between axiology and T.K.

Now, on the values that were explored, far from being interesting the differences in the frequency with which they appeared in the coding, it seems relevant to point out that in the network elaborated on this topic (Figure 8) it is possible to weave a relationship between an epistemic value such as precision and a bioethical value such as honesty through the cognitive abilities of self-control and introspection, which in turn are associated with other cognitive abilities such as self-organization and willingness to learn, respectively.

The following quote takes account of the elements that the American Academy of Pediatrics (AAP) identifies as relevant in teaching and advising pediatrics the doctor-patient relationship, which are related to skills that the physician must develop for themselves and which are associated with the skills described in the network of Figure 8:

“…the other two (self-improvement and self-awareness and knowledge of limits) relate to physician singular skills. These components must be worked on throughout medical education and on an ongoing process of continuing education after graduation” (43).

The value of precision is revealed insofar as education and discussion focused on real issues and scenarios can help healthcare personnel to better identify, distinguish and manage emotional burdens and difficult situations, as expressed in the following quote:

“Some of the contributing factors that were identified were inherent to the conflict situation and could not be tackled by direct intervention, such as emotional load, critical or precipitated situations and uncertain prognosis, although healthcare professionals can be prepared to cope with them through discussions and education” (48).

#### Epistemic and Ethical Values as Vectors to Express Cognitive Abilities

In the axiological horizon of ethical-tacit knowledge in medical practice there is an intrinsic relationship between epistemic and ethical values with cognitive skills, this means that only professionals who practice honesty, authenticity, and self-control are capable of seeing patients as persons and thus respect their dignity. This means that they develop medicine of excellence with two feet, evidence-based medicine, which has to do with cognitive abilities and epistemic values, and value-based medicine, which focuses on the patient and healthcare professionals as persons. This confirms the findings of several authors where ethical values and the role of co-partner in the doctor-patient relationship are the amalgams of the MBE-MBV binomial (7, 18, 61, 62).

#### Tacit Knowledge Skills

Regarding the three different dimensions in tacit knowledge skills -cognitive, social, and technical- there was no difference in the frequency with which they were coded, but there was a difference in the relationships that were found between them and each one with the schools of ethical thought and axiology.

**Figure 9:**
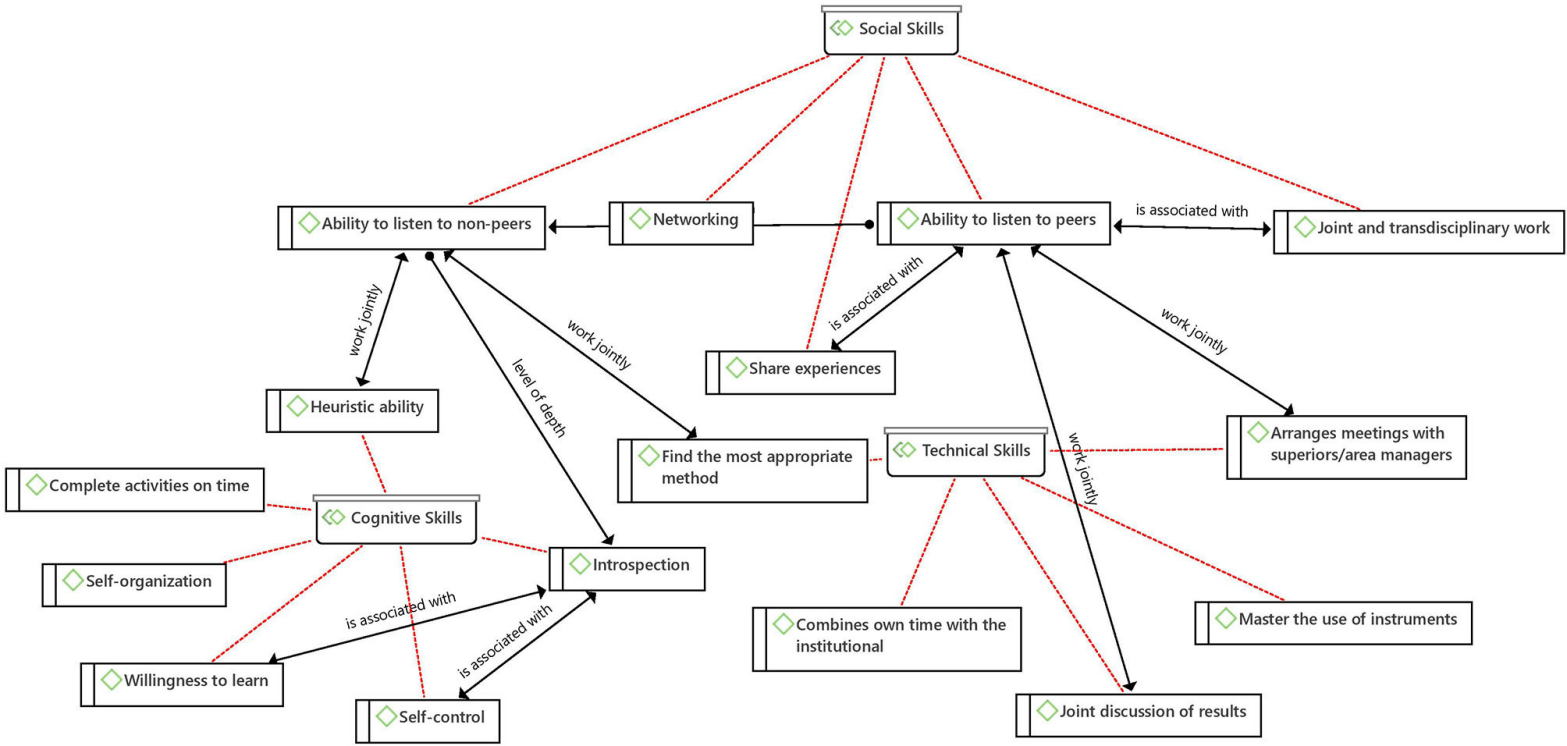
Tacit knowledge net.

With regard to tacit social knowledge, sharing experiences and the ability to listen to non-peers are of utmost importance. The ability to listen to non-peers works in conjunction with heuristic ability—cognitive skill- and with finding the most appropriate method—technical skill (Figure 9). The first refers to identifying and creating the pieces for the resolution of a problem by taking information not only from one's own baggage but also by listening to patients, which adds to other pieces of information -the patient's work and home environment, and their occupation- to find the most appropriate way to address a problem.

Although none of the social skills was directly related to any of the epistemic or bioethical values, it was possible to establish a relationship with the deontological school of ethical thought; a more structured curriculum in ethics and professionalism in the training of the medical professional is assumed as a duty but looking for accordance between what is taught in the classroom and what is learned in practice:

“Even though evolving training program requirements emphasize a more structured curriculum in ethics and professionalism, role-modeling will remain an essential teaching tool (deliberately or not), and training programs will need to resolve the dissonance between what trainees are taught and what they see” (40)

Among the three most relevant cognitive skills are heuristics and introspection, along with the willingness to learn. The last two relate to self-control, thus they operate at a level of self-awareness and search for personal improvement, skills expected to be promoted even once the doctors have graduated (43).

In previous paragraphs, the relationship between cognitive skills and the epistemic value of precision was discussed, but it is also worth mentioning the relationships of these skills with the bioethical values of honesty and respect, and with the deontological school of ethical thought, all of which can be identified in the sequence of steps described by Loren et al. (47) to reveal a medical error:

“A growing number of hospitals and pediatric medical centers and 1 professional physician organization (American College of Emergency Physicians) have adopted error disclosure policies in an effort to guide physician behavior regarding disclosure of medical errors. These policies in general recommend (1) an open and honest description of the events as soon as possible, (2) a sincere apology that recognizes the harm that occurred, (3) identification of the processes that led to the occurrence, and (4) a description of what was learned from the situation (e.g., a description of the steps that will be taken to prevent the event from recurring).”

Continuing with the type of tacit knowledge skills, as for the technical ones, the most frequent was to find the most appropriate method, followed by the joint discussion of results. The first works in conjunction with the ability to listen to non-peers—a social ability. In the developed networks it was not found that technical tacit knowledge had any relationship with epistemic or bioethical values.

#### Emerging Dilemmas and the Role of Trust in Their Outcome

As mentioned in the methodology section, at the time of coding two codes emerged as relevant, the value of trust and dilemmas. The first was found to be key to achieving communication with non-peers:

“Creating trust and using a communicative style that allows knowledgeable information to emerge and be identified seemed to be a cornerstone in the jigsaw puzzling. Here, the physicians used tacit knowledge, obtained through several encounters and also personal experiences from professional and private settings. In addition, they used explicit knowledge (e.g. medical procedures, record information) and in particular, they underscored the importance of enough time for the consultation” (56).

On the other hand, it was deemed necessary to extract, especially from Cook's work, which dilemmas are regarded in pediatrics as:

a) Frequent, but it is appreciated that they are addressed incorrectly, such as health care disparities, disabilities, conflicts of interest, ethics of genetic testing/screening, conduct on social media sites, and responding to requests for prescriptions by family, friends, or colleagues.
b) Uncommon and not addressed in education, such as conscience clauses for physicians, expert witness testimony, and enhancement therapies.
c) Those that young pediatricians are unprepared to face, such as patient/family boundaries, admitting mistakes, and addressing unprofessional behaviors and attitudes.

#### Tacit Knowledge Advantage in the Context of Covid-19

Since the COVID-19 declaration as a pandemic, its complexity has increased. This has posed an uncertain picture, with dizzying changes, in which the knowledge acquired by health personnel becomes a value-added knowledge that needs to be shared. This knowledge refers above all to the sphere of experiences, for this reason, it is mostly tacit; in order to be shared, it must be first codified and then standardized (63). Because it can be of great help both to carry out diagnoses and treatment plans in a different way (virtual) (64) and because it forces to improve the management of shared decisions (65), it is a type of knowledge that has the ability to promote both evidence-based medicine and patient-centered medicine. It can be conducted either at an individual level (63) or a collective one by strengthening the competencies to work with colleagues as a team, both because it reduces ambiguities (63) and because of the possibility of alleviating the psychological burden involved in treating patients for Covid-19 (66). Psychosocial support and enjoying free time are also fundamental (66), both of which bibliotherapy includes as a way of reflecting and re-signify own actions to extract knowledge that can be capitalized in this very particular context (67).

## Discussion

In summary, taking into consideration the three themes (ethics, axiology, and tacit knowledge) into which the analysis of the results was sub-divided and with a view to building an idea of ethical tacit knowledge, it is possible to appreciate that while ethics justifies actions insofar as they perform desirable values, according to preferable ends, ethical tacit knowledge examines from personal experience (observe or carry out a practice) the actions that define the foundations of moral judgments (values and virtues). This type of knowledge is acquired and manifested at a particular moment, “on the fly,” next to the patient's bed, with health personnel and with family members, in such a way that it is amalgamated with explicit or formal knowledge. This is how the resources from which the heuristics support decision-making, the discernment of ethical dilemmas arising in the COVID-19 pandemic, and strengthening of the binomial values and evidence-based medicine are generated.

In order to achieve this aim, we have found that there are two processes, intricately related, of greatest relevance that penetrate the three skills dimensions in which tacit knowledge operates—cognitive, technical, and social- which need with urgency to be developed in health care personnel in the pandemic: experience in terms of sharing it and the development of communication skills at different levels.

### Experience as the Range of Possibilities for Professional Growth

**Figure 10:**
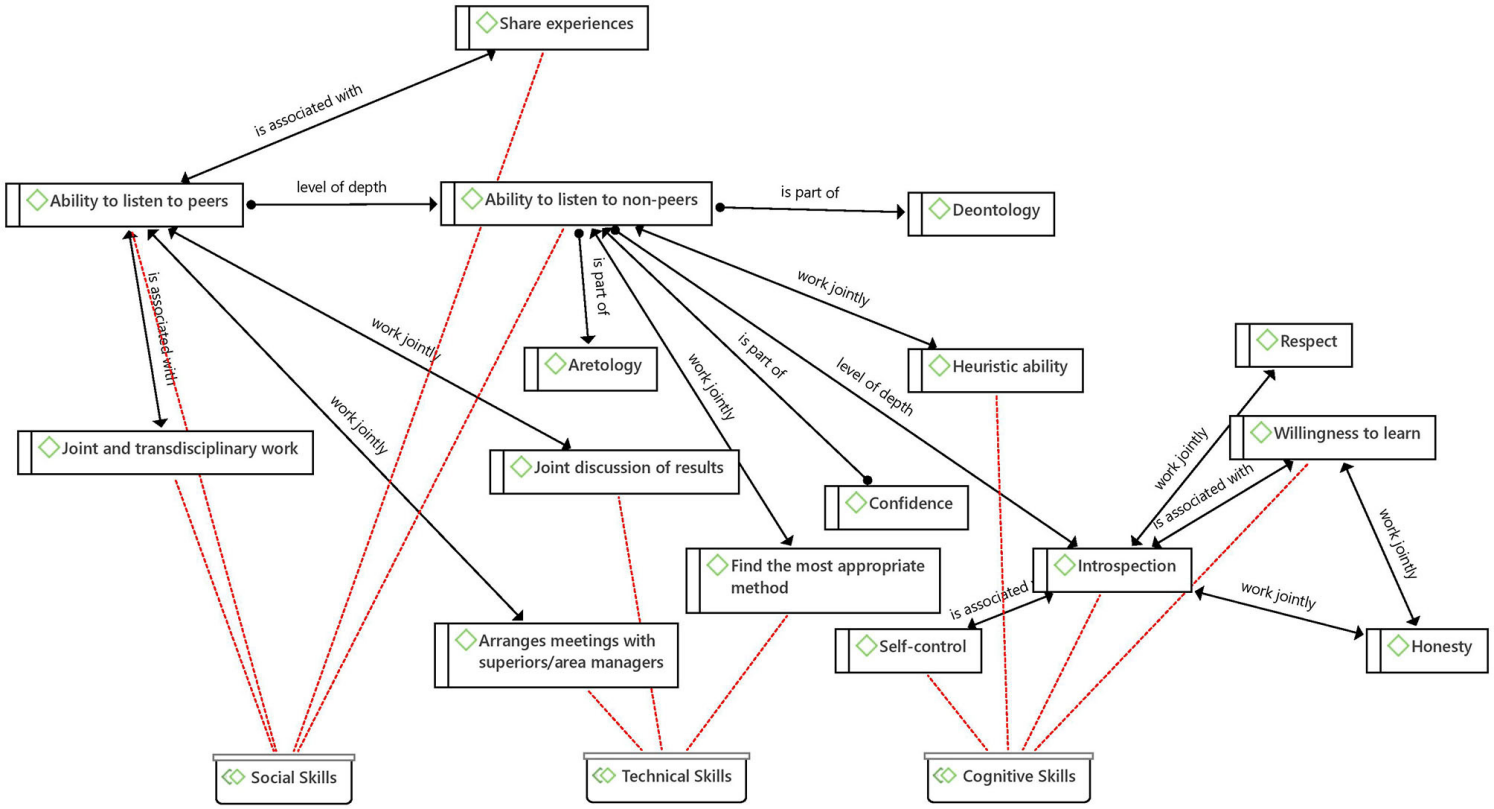
The network of experience.

The role that experience plays in the acquisition of tacit knowledge is of utmost importance, not only in a personal way—as described by Polanyi- that is, being the protagonist of the experience, but sharing experiences seems to promote the relationship between the different types of skills of tacit knowledge (Figure 10). Strengthening the ability to listen to peers and non-peers enables us to reach technical and cognitive skills, as it brings about a wide spectrum of pieces of information to use in decision-making, opening the door to a wealth of skills. We could even dare to extrapolate the incidence of this ability to the exercise of empathy and to maintain the mental health equilibrium.

But experience can only be shared if a process of reflection has been carried out beforehand to explore one's own behaviors, which in turn has as a consequence in the development of good judgment.

Although at his time Polanyi (20) expressed that “we know more than we can say” since there is a part that remains tacit in our actions, subsidiary to the focal act, it is possible to apprehend those aspects by carrying out, after the action, a communication process in different levels and (from the development of skills) guided by reflection.

### Levels of Depth in Communication

From the results, it was possible to identify a relationship between the ability to listen to non-peers, the ability to listen to peers, and introspection (interior). All three are part of different levels of communication that are exercised (a) with others whom we consider different and distant from us, (b), with others who we consider equal and therefore we value their opinions, and (c) with ourselves at a thoughtful and critical level.

**Figure 11:**
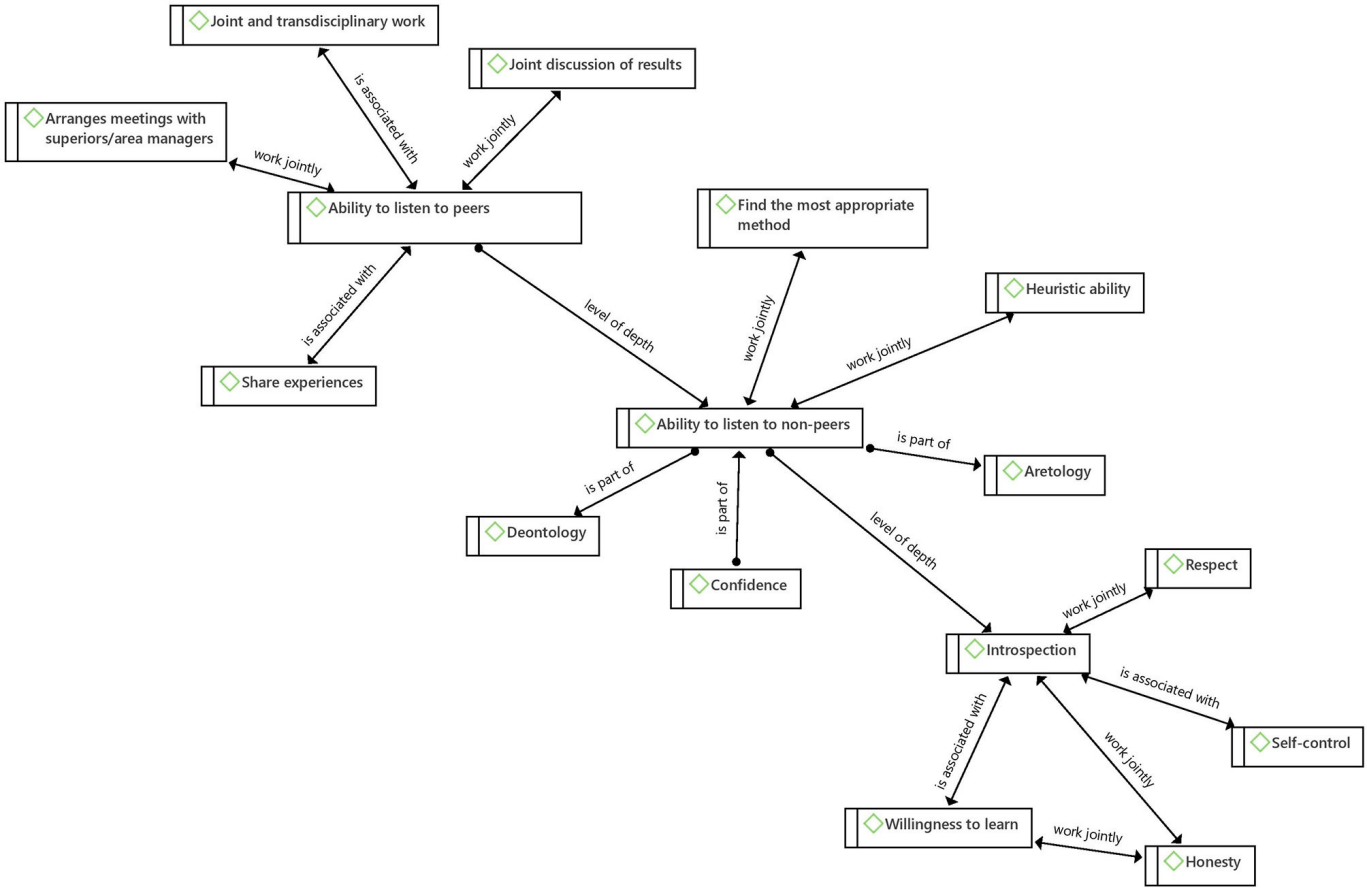
Levels of depth in communication.

The development of each of these communication skills provides at the same time the development of other skills and values such as joint discussion of results, joint and transdisciplinary work, self-control, willingness to learn, share experiences, arrange meetings with superiors/area managers, find the most appropriate method and heuristic ability (Figure 11). Further, each level of depth in communication implies a different exercise of reflection and construction of knowledge, although they constantly feedback and promote each other, and have the capacity to allow physicians to alleviate the pandemic stress.

### Tacit Knowledge and COVID-19

It should be noted that to date there are few articles that consider tacit knowledge in the context of the Covid-19 pandemic in the practice of health specialists (66).

Although the COVID-19 pandemic has represented a huge challenge for all humanity, in particular with regard to health professionals, it has forced the health specialists to rethink their practice on (1) the limits of the duty to treat or to provide medical care correctly (for instance when their lives are at risk because appropriate conditions do not exist) (68) about (2) new ways of establishing a bond with the patient in virtual care, attending to the needs of the patient in times of uncertainty (65), and (3) how to deal with the psychosocial burden of care and the need for support resources (66, 67).

In this sense, specifically with regard to tacit knowledge in terms of the context of the COVID-19 pandemic, the challenges refer to (1) adapting and learning a new way of establishing trust with the patient as well as a new way of using resources to perform diagnosis-even efficiency will take a new meaning (64), and (2) what and how to learn from the whole new experiences the pandemic has posed-the challenge is to capitalize on this new knowledge (63).

Although the present research describes a systematic review and hermeneutic analysis of the bibliography, that helps to have a broad perspective on tacit knowledge, it is to be recognized that to deepen the topic it is necessary to carry out a qualitative study that incorporates not only interviews but also a shadow observation, which will be the purpose of later work. The proposition of an educational strategy to capitalize ethical tacit knowledge in any circumstance, but especially during an emergency, such as a pandemic, will also be the purpose of later work.

### Conclusion

Before concluding by emphasizing the challenges in teaching-learning of ethical tacit knowledge, it should be noted that in his time, Polanyi (69) left guidelines not only to explore how knowledge is built, denoting the relevance of its tacit dimension but also dealt, although very briefly, on how the tacit framework of moral acts and judgments is established through internalization -identifying ourselves with the teachings in question. As Amartya Sen stated in the preface to Polanyi's book “The tacit dimension” (69) all knowledge is internalized insofar as it is disclosed as relevant in the world in which we carry out our daily routines amid the COVID-19 pandemic, that is, it belongs to a context and is at the service of the reality to which we submit.

This is an aspect that any educational strategy must consider whether it is based on problem-solving or a video reflexive ethnography, as our findings show. Examples used to generate discussion and subsequent reflection need to be grounded in the closest reality of the students, in this case, medical personnel, so that their usefulness is fully perceived and therefore knowledge be internalized, becoming tacit.

Another result of the revision of tacit knowledge, in its ethical aspects, shows that it is not sufficient to teach values in a theoretical way to learn to discern when to put them into practice in concrete actions and scenarios, that is, it is necessary to accompany them with the development of different types of skills.

Ideal educational strategies should focus on dialogue and discussion to promote introspection and the other levels of depth in communication mentioned above, in order to extract the most from experiences and build an ethical tacit knowledge that responds to the needs of health personnel in any of their specialties and any context, such as a pandemic. This will be a vein that needs to be explored and exploited for the benefit of clinical practice and patients.

Although no references were found that dealt with any of the main topics -ethics, axiology, and tacit knowledge- in pediatric endocrinology, there is an interest in pediatrics to explore and discuss educational strategies in ethics related to its tacit dimension.

## Data Availability Statement

The original contributions presented in the study are included in the article/Supplementary Material, further inquiries can be directed to the corresponding author/s.

## Author Contributions

All authors listed have made a substantial, direct, and intellectual contribution to the work and approved it for publication.

## Funding

The authors declare that this study received funding from a Ph.D. fellowship from the Mexican National Council for Science and Technology CONACyT (366631). The funder was not involved in the study design, collection, analysis, interpretation of data, the writing of this article, or the decision to submit it for publication.

## Conflict of Interest

The authors declare that the research was conducted in the absence of any commercial or financial relationships that could be construed as a potential conflict of interest.

## Publisher's Note

All claims expressed in this article are solely those of the authors and do not necessarily represent those of their affiliated organizations, or those of the publisher, the editors and the reviewers. Any product that may be evaluated in this article, or claim that may be made by its manufacturer, is not guaranteed or endorsed by the publisher.
