# Supplementary material for: Appealing to Tacit Knowledge and Axiology to Enhance Medical Practice in the COVID-19 Pandemic: A Systematic Review and Hermeneutic Bioethical Analysis
Source: Front Public Health. 2021 Dec 8;9:686773. doi: 10.3389/fpubh.2021.686773 (PMC8692268; doi:10.3389/fpubh.2021.686773)
Supplement: Supplementary file 1 [file Table_1.DOCX]

Supplementary Material

# Supplementary Data

**SP 1.** Precise description of the search results of the MeSH terms in PubMed

**PARTICIPANTS**

**Medicine = 6,070,593 results**

"medicin"[All Fields] OR "medicinal"[All Fields] OR "medicinally"[All Fields] OR "medicinals"[All Fields] OR "medicine"[MeSH Terms] OR "medicine"[All Fields] OR "medicine s"[All Fields] OR "medicines"[All Fields]

**“Clinical practice” = 188,349 results**

"clinical practice"[All Fields]

**Medicine OR “Clinical practice” = 6,183,237**

"medicin"[All Fields] OR "medicinal"[All Fields] OR "medicinally"[All Fields] OR "medicinals"[All Fields] OR "medicine"[MeSH Terms] OR "medicine"[All Fields] OR "medicine s"[All Fields] OR "medicines"[All Fields] OR "clinical practice"[All Fields]

**Physician = 643,310 results**

"physician s"[All Fields] OR "physicians"[MeSH Terms] OR "physicians"[All Fields] OR "physician"[All Fields] OR "physicians s"[All Fields]

**“Medical doctor” = 1,618 results**

"medical doctor"[All Fields]

**Physician OR “Medical doctor” = 644,504 results**

"physician s"[All Fields] OR "physicians"[MeSH Terms] OR "physicians"[All Fields] OR "physician"[All Fields] OR "physicians s"[All Fields] OR "medical doctor"[All Fields]

**Pediatrician = 24,406 results**

"paediatrician s"[All Fields] OR "paediatricians"[All Fields] OR "pediatrician s"[All Fields] OR "pediatricians"[MeSH Terms] OR "pediatricians"[All Fields] OR "paediatrician"[All Fields] OR "pediatrician"[All Fields]

**Endocrinologist =** **5,047 results**

"endocrinologist s"[All Fields] OR "endocrinologists"[MeSH Terms] OR "endocrinologists"[All Fields] OR "endocrinologist"[All Fields]

**Pediatrician OR Endocrinologist =** **29,260 results**

"paediatrician s"[All Fields] OR "paediatricians"[All Fields] OR "pediatrician s"[All Fields] OR "pediatricians"[MeSH Terms] OR "pediatricians"[All Fields] OR "paediatrician"[All Fields] OR "pediatrician"[All Fields] OR "endocrinologist s"[All Fields] OR "endocrinologists"[MeSH Terms] OR "endocrinologists"[All Fields] OR "endocrinologist"[All Fields]

**(((medicine) OR ("clinical practice")) AND ((physician) OR ("medical doctor"))) AND ((pediatrician) OR (endocrinologist)) = 6,466 results**

((((((((("medicin"[All Fields] OR "medicinal"[All Fields]) OR "medicinally"[All Fields]) OR "medicinals"[All Fields]) OR "medicine"[MeSH Terms]) OR "medicine"[All Fields]) OR "medicine s"[All Fields]) OR "medicines"[All Fields]) OR "clinical practice"[All Fields]) AND ((((("physician s"[All Fields] OR "physicians"[MeSH Terms]) OR "physicians"[All Fields]) OR "physician"[All Fields]) OR "physicians s"[All Fields]) OR "medical doctor"[All Fields])) AND ((((((("paediatrician s"[All Fields] OR "paediatricians"[All Fields]) OR "pediatrician s"[All Fields]) OR "pediatricians"[MeSH Terms]) OR "pediatricians"[All Fields]) OR "paediatrician"[All Fields]) OR "pediatrician"[All Fields]) OR ((("endocrinologist s"[All Fields] OR "endocrinologists"[MeSH Terms]) OR "endocrinologists"[All Fields]) OR "endocrinologist"[All Fields]))

**EXPOSITION / PARTICIPANTS + EXPOSITION**

**Survey =** **1,460,809 results**

(((((("survey s"[All Fields] OR "surveyed"[All Fields]) OR "surveying"[All Fields]) OR "surveys and questionnaires"[MeSH Terms]) OR ("surveys"[All Fields] AND "questionnaires"[All Fields])) OR "surveys and questionnaires"[All Fields]) OR "survey"[All Fields]) OR "surveys"[All Fields]

**Interview = 207,608 results**

"interview"[Publication Type] OR "interviews as topic"[MeSH Terms] OR "interview"[All Fields]

**“Participant observation” = 3,482 results**

"participant observation"[All Fields]

**((survey) OR (interview)) OR ("participant observation") = 1,605,670**

(((((((("survey s"[All Fields] OR "surveyed"[All Fields]) OR "surveying"[All Fields]) OR "surveys and questionnaires"[MeSH Terms]) OR ("surveys"[All Fields] AND "questionnaires"[All Fields])) OR "surveys and questionnaires"[All Fields]) OR "survey"[All Fields]) OR "surveys"[All Fields]) OR (("interview"[Publication Type] OR "interviews as topic"[MeSH Terms]) OR "interview"[All Fields])) OR "participant observation"[All Fields]

**((((medicine) OR ("clinical practice")) AND ((physician) OR ("medical doctor"))) AND ((pediatrician) OR (endocrinologist))) AND (((survey) OR (interview)) OR ("participant observation")) = 2,578 results**

(((((((((("medicin"[All Fields] OR "medicinal"[All Fields]) OR "medicinally"[All Fields]) OR "medicinals"[All Fields]) OR "medicine"[MeSH Terms]) OR "medicine"[All Fields]) OR "medicine s"[All Fields]) OR "medicines"[All Fields]) OR "clinical practice"[All Fields]) AND ((((("physician s"[All Fields] OR "physicians"[MeSH Terms]) OR "physicians"[All Fields]) OR "physician"[All Fields]) OR "physicians s"[All Fields]) OR "medical doctor"[All Fields])) AND ((((((("paediatrician s"[All Fields] OR "paediatricians"[All Fields]) OR "pediatrician s"[All Fields]) OR "pediatricians"[MeSH Terms]) OR "pediatricians"[All Fields]) OR "paediatrician"[All Fields]) OR "pediatrician"[All Fields]) OR ((("endocrinologist s"[All Fields] OR "endocrinologists"[MeSH Terms]) OR "endocrinologists"[All Fields]) OR "endocrinologist"[All Fields]))) AND ((((((((("survey s"[All Fields] OR "surveyed"[All Fields]) OR "surveying"[All Fields]) OR "surveys and questionnaires"[MeSH Terms]) OR ("surveys"[All Fields] AND "questionnaires"[All Fields])) OR "surveys and questionnaires"[All Fields]) OR "survey"[All Fields]) OR "surveys"[All Fields]) OR (("interview"[Publication Type] OR "interviews as topic"[MeSH Terms]) OR "interview"[All Fields])) OR "participant observation"[All Fields])

**OUTCOME / PARTICIPANTS + EXPOSITION + OUTCOME**

**“Tacit knowledge” = 446 results**

"tacit knowledge"[All Fields]

**Ethics = 345,204 results**

"ethic s"[All Fields] OR "ethicality"[All Fields] OR "ethically"[All Fields] OR "ethics"[MeSH Terms] OR "ethics"[All Fields] OR "ethic"[All Fields] OR "ethics"[MeSH Subheading] OR "morals"[MeSH Terms] OR "morals"[All Fields] OR "ethical"[All Fields]

**Axiology = 48 results**

"axiology"[All Fields]

**(("tacit knowledge") OR (Ethics)) OR (axiology) = 345,641 results**

"tacit knowledge"[All Fields] OR "ethic s"[All Fields] OR "ethicality"[All Fields] OR "ethically"[All Fields] OR "ethics"[MeSH Terms] OR "ethics"[All Fields] OR "ethic"[All Fields] OR "ethics"[MeSH Subheading] OR "morals"[MeSH Terms] OR "morals"[All Fields] OR "ethical"[All Fields] OR "axiology"[All Fields]

**(((((medicine) OR ("clinical practice")) AND ((physician) OR ("medical doctor"))) AND ((pediatrician) OR (endocrinologist))) AND (((survey) OR (interview)) OR ("participant observation"))) AND ((("tacit knowledge") OR (Ethics)) OR (axiology)) = 128 results**

((((((((((("medicin"[All Fields] OR "medicinal"[All Fields]) OR "medicinally"[All Fields]) OR "medicinals"[All Fields]) OR "medicine"[MeSH Terms]) OR "medicine"[All Fields]) OR "medicine s"[All Fields]) OR "medicines"[All Fields]) OR "clinical practice"[All Fields]) AND ((((("physician s"[All Fields] OR "physicians"[MeSH Terms]) OR "physicians"[All Fields]) OR "physician"[All Fields]) OR "physicians s"[All Fields]) OR "medical doctor"[All Fields])) AND ((((((("paediatrician s"[All Fields] OR "paediatricians"[All Fields]) OR "pediatrician s"[All Fields]) OR "pediatricians"[MeSH Terms]) OR "pediatricians"[All Fields]) OR "paediatrician"[All Fields]) OR "pediatrician"[All Fields]) OR ((("endocrinologist s"[All Fields] OR "endocrinologists"[MeSH Terms]) OR "endocrinologists"[All Fields]) OR "endocrinologist"[All Fields]))) AND ((((((((("survey s"[All Fields] OR "surveyed"[All Fields]) OR "surveying"[All Fields]) OR "surveys and questionnaires"[MeSH Terms]) OR ("surveys"[All Fields] AND "questionnaires"[All Fields])) OR "surveys and questionnaires"[All Fields]) OR "survey"[All Fields]) OR "surveys"[All Fields]) OR (("interview"[Publication Type] OR "interviews as topic"[MeSH Terms]) OR "interview"[All Fields])) OR "participant observation"[All Fields])) AND (("tacit knowledge"[All Fields] OR ((((((((("ethic s"[All Fields] OR "ethicality"[All Fields]) OR "ethically"[All Fields]) OR "ethics"[MeSH Terms]) OR "ethics"[All Fields]) OR "ethic"[All Fields]) OR "ethics"[MeSH Subheading]) OR "morals"[MeSH Terms]) OR "morals"[All Fields]) OR "ethical"[All Fields])) OR "axiology"[All Fields])

# Supplementary Figures and Tables

## Supplementary Tables

**
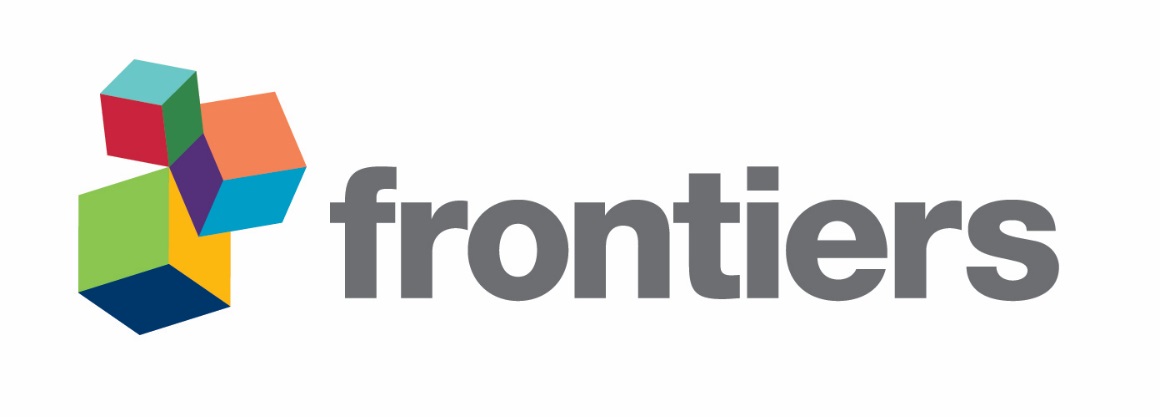
**

**SP 2** Quantitative information from articles about Ethics Theme

| **Author/Year** | **Setting** | **Main school (s) of thought or concepts** | **Study design** | **Target population** | **Epistemic subject** | **Quality index** |
| --- | --- | --- | --- | --- | --- | --- |
| Larson/2017 | Canada | Moral distress, burnout, uncertainty, perceived hospital supportiveness | Cross-sectional survey | Neonatal and health practitioners | There is a need to meet own and others' expectations, this may differ depending on experience and the role played | 2 |
| Placencia/2016 | United States | Best Interests Standard (BIS), end-of-life decision making | Survey | Neonatologists and bioethicists | When conceptual consensus is not shared with other, that may result in different ways of making judments and taking decisions | 2 |
| Grosek/2016 | Slovenia | Patient´s best interest, good clinical practice, patient’s autonomous decision, paternalistic approach | Cross-sectional survey | Paediatric intensivists, paediatric specialists and paediatric residents | Learns to make decisions from the experience of practice and education | 2 |
| Morrison/2015 | United States | Ethical assistance, mediation skills | Survey | Physician members of the American Academy of Pediatrics | Needs appropriate training and experience in clinical ethics | 1 |
| Cook/2013 | United States | Structured ethics curriculum, role-modeling | Survey | Young pediatricians | Role-modeling (single observation) is insufficient as the sole approach to learn values | 3 |
| Todres/2000 | United States | Professional autonomy, quality of life | A long-term follow-up survey | Pediatricians | Specific patient care experiences are considered an influence in changing attitudes | 1 |
| Bucher/2018 | Switzerland | Withholding or withdrawing intensive care, shared decision-making, diverse moral attitudes and values | Survey | Neonatologists and neonatal nurses | Acknowledging differences in ethics education, patient care focus and exposure to the patients may help to avoid conflicts when taking decisions | 2 |
| Kesselheim/2008 | United States | Confidence confronting ethical challenges, informal discussion | Cross-sectional survey | Physicians who completed pediatric or medicine/pediatric residency | Confidence to adress challenges is reached with others support and discussions | 3 |
| Guedert/2012 | Brazil | Formal and hidden curriculum to deal with ethical problems, virtue-based ethics | Self-applied semi-structured questionnaire | Physicians with teaching and pediatric care responsibilities | Constant reflection on professional attitudes and ethical issues is necessary due to the diversity of circumstances | 3 |
| Farre/2016 | United Kingdom | Healthcare Information Technology (HIT), informal work practice, social and organisational contextual factors | Longitudinal qualitatively driven mixed- method approach | Paediatricians, pediatric nurses, pediatric pharmacists,pharmacy technicians and managers | Incorporating a device to individual everyday practice is a complex social process | 1 |
| Lynöe/1998 | Sweden | Ethical reasoning, tension between the principle of the autonomy of the individual patient and a paternalistic attitude | Questionnaire | General practitioners, gynaecologists, and pediatricians | Ethical motives (according to the background profile) are the basis of doctor´s attitude in problem solving | 1 |
| Irwig/2018 | United States | Conflicts of interest, clinical practice guidelines | Self-reports and Open data | Clinical Practice Guidelines published in The Journal of Clinical Endocrinology & Metabolism | People cannot take clinical practice guidelines as reliable on their own, they must consider the context of their preparation | 1 |
| Cotter/2019 | United States | Effective communication, reflective practice | Implementation of an escalation curriculum with pre and post surveys | Senior pediatric residents | The process of reflexion maximizes learning from experience | 3 |
| Goggin/2018 | United States | Parents-provider communication, perception of expectations, shared decision-making | Educational interventions and surveys | Paediatricians, nurse practitioners and parent–child dyads | The development of communication skills is of outmost relevance to provide and receive satisfactory care | 2 |

**SP 3.** Quantitative information from articles about Axiology Theme

| **Author/Year** | **Setting** | **Theoretical axis** | **Study design** | **Target population** | **Quality index** |
| --- | --- | --- | --- | --- | --- |
| Loren/2008 | United States | Error disclousure, truth telling | Survey | Pediatricians and pediatric residents | 3 |
| Smith/2007 | United States | Overall career satisfaction | Survey: The Physician Values in Practice Scale | Recently graduated medicine students | 2 |
| Archambault/2018 | Canada | Peer discussion in decision-making process, ethical confrontation | Questionnaire | Pediatric staff | 3 |
| Domínguez/2012 | Spain | Teamwork, intersectorality | Statistics review | Primary care pediatrics | 1 |
| Szawarski/1988 | Poland | Respect for life, paternalistic role, decision making by physicians | Questionnaire from an australian research group | Pediatricians at neonatal and intensive-care departments | 2 |

**SP 4.**Quantitative information from articles about Tacit knowledge Theme

| **Author/Year** | **Setting** | **Theoretical axis** | **Study design** | **Target population** | **Tacit knowledge conception** | **Tacit Knowledge relevance** | **Fields related to Tacit knowledge** | **Quality index** |
| --- | --- | --- | --- | --- | --- | --- | --- | --- |
| Kothari/2012 | Canada | Knowledge value chain | Narrative inquiry | Public Health practitioners | Mc Adams *et al.* :"the knowledge in practice developed from direct experience and action; highly pragmatic and specific according to the situation; subconsciously understood and applied; difficult to articulate; commonly shared through interactive conversation and shared experience" | To help in the planning of programs | Public health | 3 |
| Thornton/2006 | United Kingdom | Practical *know-how* | Theoretical discussion-analysis | Clinical practice | Implicit knowledge | For the development of good judgment | Clinical practice in general | 1 |
| Cruess/2014 | Canada | Value system, professional identity | Theoretical discussion-analysis | Medicine students | Hidden elements and an unconscious level of learning and construction of being | It is a component of the professional identity | Medicine in general | 2 |
| Henry/2006 | United States | Intuition | Theoretical discussion-analysis | Medical Practice | They are those aspects of human knowledge that function in a subsidiary and non-specific manner in the periphery of attention and that make possible the conventional explicit dimensions | To take qualitative knowledge as relevant | Clinical encounter, medical decision making | 2 |
| Braude/2009 | Canada | Intuition | Theoretical discussion-analysis | Clinical epidemiology | Analogous to practical intuition, it is the combination of the subsidiary and corporal poles | To re-conceptualize the cognition of clinical reasoning | Clinical intuition, clinical epidemiology | 1 |
| Ringstad/2014 | Norway | Autonomous decision | Semi-structured interviews | Patient, nurse, physioterapist and physician of a rehabilitation team | It is a kind of learning that includes practical and bodily knowledge that is not expressed verbally or stated directly | Seen as personal experience it is a complementary element for understanding a specific situation | Clinical rehabililtation | 3 |
| White/2006 | United States | Diagnostic and judgment process | Theoretical discussion-analysis | Clinicians | A subconscious form of knowledge, born of experience, enabling one person understand another, based on the smallest of cues; we usually do not verbalize it because we are not aware we have it | Tacit knowledge and intuition may provide valuable diagnostic evidence | Neurology | 2 |
| Bertilsson/2018 | Sweden | Process of explicitation of tacit knowledge, physician-patient encounter | Open-ended interviews and presentation of a short video vignette | Physicians specialized in general practice, occupational health or psychiatry | A kind of knowledge that is considered to be experience-based and practical, obtained through practice and repeated actions | Tacit knowledge can be a way to assess whether you have a skill; experience as a source for learning | Psychiatry | 3 |
| Majima/2018 | Japan | Reflexion upon own practices | Video reflexive ethnography | Clinical nursing | Practical knowledge | Skills education for the discovery and sharing of practical knowledge | Nursing | 1 |
| Cheah/2003 | Malasia | Problem-based learning (PBL) | PBL-healthcare scenarios-based | Medicine students | It is non-formalized knowledge, which governs expert skills, common sense and intuitive judgment | To improve problem-based learning | Health care (learning) | 1 |
| Nakken/2018 | Denmark | Physician-patient communication | Semi-structured interviews | Three doctors and three public health nurses | Knowledge which originates in experience and is not systematized | To elaborate guides about the tactics employed to extract information when interviewing a patien | Public health | 2 |
